# Microglial Activation Correlates with Disease Progression and Upper Motor Neuron Clinical Symptoms in Amyotrophic Lateral Sclerosis

**DOI:** 10.1371/journal.pone.0039216

**Published:** 2012-06-14

**Authors:** Johannes Brettschneider, Jon B. Toledo, Vivianna M. Van Deerlin, Lauren Elman, Leo McCluskey, Virginia M.-Y. Lee, John Q. Trojanowski

**Affiliations:** 1 Center for Neurodegenerative Disease Research (CNDR), University of Pennsylvania School of Medicine, Philadelphia, Pennsylvania, United States of America; 2 Department of Pathology and Laboratory Medicine, University of Pennsylvania School of Medicine, Philadelphia, Pennsylvania, United States of America; 3 Department of Neurology, University of Pennsylvania School of Medicine, Philadelphia, Pennsylvania, United States of America; 4 Department of Neurology, University of Ulm, Ulm, Germany; Mayo Clinic, United States of Amercia

## Abstract

**Background/Aims:**

We evaluated clinicopathological correlates of upper motor neuron (UMN) damage in amyotrophic lateral sclerosis (ALS), and analyzed if the presence of the *C9ORF72* repeat expansion was associated with alterations in microglial inflammatory activity.

**Methods:**

Microglial pathology was assessed by IHC with 2 different antibodies (CD68, Iba1), myelin loss by Kluver-Barrera staining and myelin basic protein (MBP) IHC, and axonal loss by neurofilament protein (TA51) IHC, performed on 59 autopsy cases of ALS including 9 cases with *C9ORF72* repeat expansion.

**Results:**

Microglial pathology as depicted by CD68 and Iba1 was significantly more extensive in the corticospinal tract (CST) of ALS cases with a rapid progression of disease. Cases with *C9ORF72* repeat expansion showed more extensive microglial pathology in the medulla and motor cortex which persisted after adjusting for disease duration in a logistic regression model. Higher scores on the clinical UMN scale correlated with increasing microglial pathology in the cervical CST. TDP-43 pathology was more extensive in the motor cortex of cases with rapid progression of disease.

**Conclusions:**

This study demonstrates that microglial pathology in the CST of ALS correlates with disease progression and is linked to severity of UMN deficits.

## Introduction

Amyotrophic lateral sclerosis (ALS) is the most frequent adult-onset motor neuron disease, characterized by the combined degeneration of the upper motor neurons (UMN) of the corticospinal tract (CST) and the lower motor neurons (LMN) of the spinal cord anterior horns, leading to death after a mean survival of approximately three years [1]. Neuronal degeneration in ALS is accompanied by the presence of hallmark ubiquitinated cytoplasmic inclusions, which were shown to be formed by the 43-kDa TAR DNA-binding protein (TDP-43) in the majority of ALS cases [2]. Genetically, ALS is mostly sporadic (sALS) but approximately 10% of cases have a first- or second-degree relative with the disease suggestive of familial ALS (fALS). Mutations in *SOD1*, encoding the Cu/Zn superoxide dismutase, *TARDBP* encoding TDP-43, fused in sarcoma (*FUS*) and the optineurin (*OPTN*) gene were observed to account for ∼30% of fALS cases [3]–[7]. Recently, a noncoding GGGGCC hexanucleotide repeat expansion in the C9ORF72 gene was identified as the most common genetic abnormality in fALS and sALS [8]–[10]. ALS and frontotemporal lobar degeneration (FTLD) cases with *C9ORF72* expansion were observed not to contain protein aggregates comprised of the C9ORF72 protein [10], [11] though TDP-43 inclusions were observed and p62 was suggested to be the major disease protein since p62-immunoreactive neuronal cytoplasmic inclusions were found in the cerebral cortex, basal ganglia, hippocampus, and cerebellum [6], [9], [12].

A major conceptual advance was the notion that ALS is not an autonomous disease of neurons, but a multiple-system disease with an important role played by astrocytes [4], [5], [13]–[15] and microglia [6], [16], [17]. Several studies demonstrated extensive microglial pathology in cases with ALS [18]–[20], and inflammatory mechanisms, including microglia, have been implicated in mediating neuronal cell death as well as promoting neuronal survival [21]–[23]. Drugs aimed at inflammatory pathways were shown to have beneficial effects on survival in transgenic mouse models of ALS [24], [25], though this has not been substantiated in human ALS clinical trials so far [26], [27]. This notwithstanding, glial cells are likely to have an impact on disease pathology in ALS that goes far beyond the notion of an unspecific response to neuronal degeneration.

Although the relevance of microglia in ALS pathology is well established, few studies have systematically related microglial pathology to the clinical phenotype of ALS [20], [28]. It is furthermore unclear how the presence of the *C9ORF72* repeat expansion affects non-neuronal cells involved in ALS pathology and if it is associated with alterations in microglial inflammatory activity. Here, we describe neuropathological findings in a large and clinically well-defined cohort of ALS and evaluate the relevance of *C9ORF72* gene mutations to microglial pathology and clinical phenotypes, focusing on motor symptoms and progression of disease.

## Methods

### Ethics Statement

The study was performed according to the provisions of the Helsinki Declaration. Written informed consent was obtained from all autopsy cases or their next of kin, and the study was approved by University of Pennsylvania Institutional Review Board (Penn IRB).

### Autopsy Cohort

Individuals who underwent autopsy in the Center for Neurodegenerative Disease Research at the University of Pennsylvania from 2004 to 2010 were enrolled. Our cohort included 59 cases with a clinical diagnosis of definite ALS in accordance with the modified El Escorial Criteria [29] and a neuropathological diagnosis of ALS (Table 1). Detailed clinical characteristics (gender, age at onset, age at death, site of onset, disease duration, ALS global disease severity as measured by a functional rating score (ALSFRS-R) [30], and the Medical Research Council Sumscore (MRCS) [31] were ascertained by retrospective chart review of clinic visits from 2004 through 2010 at the ALS Center within the University of Pennsylvania Health System; the vast majority of patients had been seen by two neurologists (L.E., L.M.). Unless otherwise specified, results of clinical testing used in this study were from the visit most proximate to the patients’ death, occurring within 3 months of death. Of the ALS cases included here, 11 (18.6%) had a family history of ALS, 12 (20.3%) had a family history of other neurodegenerative diseases, 32 (54.2%) were sporadic, and for 4 cases (6.8%) family history was unknown. The mean postmortem interval to autopsy for this cohort was 12.4 (+/−7.1 hours).

**Table 1:** Demographic data of ALS autopsy cases included in this study.

	ALS all	ALS C9+	ALS C9−	S
**N (female/male)**	**59 (19/40)**	**9 (2/7)**	**50 (17/33)**	
**Bulbar onset (n)**	18 (30.5%)	5 (55.6%)	13 (26%)	p = 0.04
	**Median (interquartile range)**			
**Age at onset [years]**	59 (52.5–67)	56 (53.8–63.3)	59 (52–68)	NS
**Age at death [years]**	62 (56–70)	59 (56.3–65.3)	63 (56–72)	NS
**Disease duration [months]**	24 (18–45)	24 (21–30)	30.6 (18–48)	NS
**ALSFRS-R**	19 (14.8–24.3)	26 (21–31.3)	19 (14–22.5)	p = 0.02
**MRCS**	39.5 (34.5–48)	45.5 (37–55)	38.5 (34–46.5)	NS
**UMN Score**	10 (2.75–17)	4 (1.5–17.8)	10.5 (4–17)	NS

ALSFRS-R  =  revised ALS functional rating scale, MRCS  =  Medical Research Council Sumscore, NS  =  not significant, UMN  =  upper motor neuron, S  =  statistical significance.

### Score to Assess UMN Involvement

To obtain a parametric scale of UMN involvement, patients were graded in terms of UMN “burden”, using a novel score that combined an assessment of spasticity based on the Ashworth Spasticity Scale [32] with reflex scoring [33], and rating of pseudobulbar affect [34]. In brief, spasticity for each extremity was rated from “0” (indicating no increase of muscle tonus) to “2” (indicating considerable increase in tone equivalent to rigidity of the extremity). In addition, three reflexes for each extremity were scored (upper extremity: biceps reflex, triceps reflex, finger flexors; lower extremity: patellar reflex, crossed adduction, ankle reflex), with the score ranging from “0” (normal or absent reflex) to “1” (pathologically brisk or retained reflex in a paretic extremity). Furthermore, the presence or absence of muscle clonus and pyramidal signs (e.g. Babinski sign, Hoffman’s sign) was scored, as was the presence or absence of pseudobulbar affect (“0” indicating absence, “1” indicating presence of these signs). This led to a comprehensive score of UMN involvement ranging from a minimum of 0 (no signs of UMN involvement) to a maximum of 32 (severe UMN involvement). A detailed description of the score is provided as supporting Table 1 (Table S1).

### Basic Neuropathological Characterization

Pathology was examined in the grey and white matter of 4 regions of the central nervous system (CNS) extending over the whole length of the neuraxis: motor cortex (precentral gyrus), medulla oblongata, cervical spinal cord (CSC), and lumbar spinal cord (LSC). For the spinal cord sections, the grey matter examined was the anterior horn and the white matter examined was the anterior and lateral portion of the CST. Sections were fixed and cut into 6–10 µm sections, stained with hematoxylin and eosin (H&E) and Thioflavin S, and immunohistochemistry (IHC) was performed with antibodies to tau, α-synuclein, ubiquitin, and TDP-43 as previously described [2], [35]–[37]. The extent of TDP-43, tau and plaque pathology as well as the extent of neuron loss (as monitored by HE) were rated for each region on a 4-point ordinal scale (0, none; 1, mild; 2, moderate; 3, severe/numerous) [38], [39].

### Analysis of Microglial Pathology and Axonal Loss

**Figure 1:**
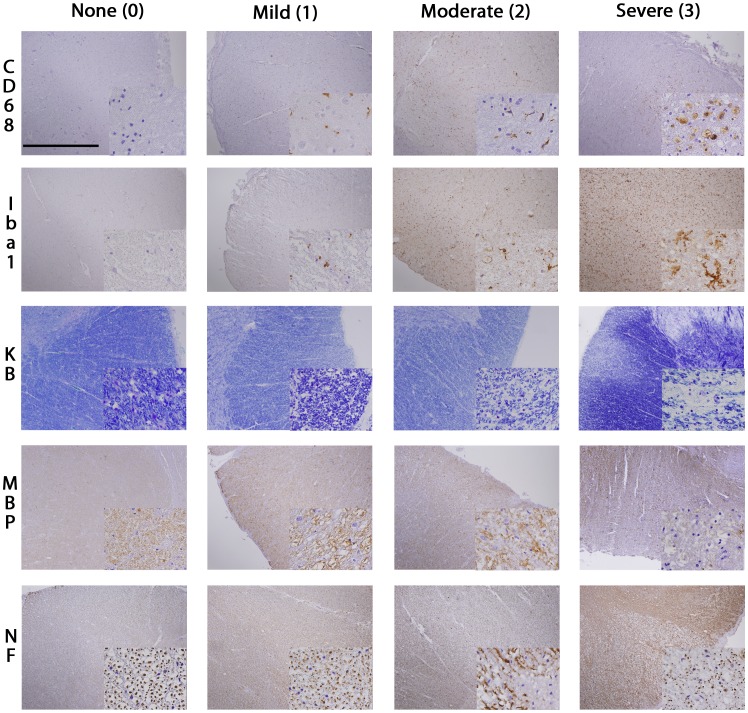
Staging of CST degeneration in ALS. The figure illustrates the IHC staging used to grade the extent of microglial activation (CD68, Iba1) and axonal loss (MBP, KB, NF) in the CST of ALS patients. Images are taken from the lateral portion of the cervical CST. KB  =  Kluver-Barrera, MBP =  myelin basic protein, NF  =  neurofilaments (TA51). Large images were taken with 4× objective, Scale bar is 1.0 mm. Small insert images were taken with 60× objective. Small insert image for CD68 stage “0” shows prominent neuronal nuclei, but no activated microglia.

Sections of 6–10 µm thickness were cut from paraffin-embedded specimens. For IHC all slides were deparaffinized and rehydrated in a series of xylene and graded ethanol. After immersion in methanol/H2O2 for 30 min, slides were washed in 0.1 M Tris buffer (pH 7.6) and blocked in 0.1 M Tris/2% FBS. Sections were stained using polyclonal rabbit anti-Iba1 antibody (Wako Chemicals, Richmond, VA) at 1∶1.000 and incubated overnight at 4°C. Sections were then rinsed and washed in Tris and incubated with Vector biotinylated anti-rabbit IgG (Vector Laboratories Inc., Burlingame, Ca) at 1∶1.000 for 1 h. After rinsing again the ICH reaction was visualized using 3,3′-diaminobenzidine (DAB) and the sections were dehydrated through graded ethanol, cleared in xylene, and coverslipped in Cytoseal 60 mounting medium. Sections were stained for CD68 using mouse anti-human CD68 (Dako, Carpinteria, CA) at 1∶1.000. The extent of microglial activation was rated for each region on a 4-point ordinal scale (0, none; 1, mild; 2, moderate; 3, severe/numerous) as previously described [38], [39] (Figure 1). Staining for myelin basic protein (MBP) was performed as described before [40], as was Kluver-Barrera (KB) staining, while IHC for the phosphorylated species of the two neurofilament heavy chains (Nf) was used to assess axonal loss in the anterior and lateral CST of these ALS subjects. Staining of Nf was performed using TA51 which recognizes a phosphorylation-dependent epitope in the carboxy terminus of the high and middle molecular weight NF subunits as described [41], [42]. The primary antibody (supernatant) was used at 1∶200. This antibody is considered highly specific of Nf and does not cross-react with other cytoskeletal proteins [15].

Double-labeling immunofluorescence (IF) analyses were performed as previously described [2] using Alexa Fluor 488- and 594-conjugated secondary antibodies (Molecular Probes, Eugene, OR), treated for autofluorescence with Sudan Black solution [43], and coverslipped with Vectashield-DAPI mounting medium (Vector Laboratories). Fluorescence images were obtained using a Leica TCS SPE-II scanning laser confocal microscope.

### Genetics Methods

Genomic DNA was extracted from peripheral blood or brain tissue following the manufacturer’s protocols (Flexigene (Qiagen, Valencia, CA) or QuickGene DNA whole blood kit L (Autogen, Holliston, MA) for blood, and QIAsymphony DNA Mini Kit (Qiagen) for brain). Genotyping for a C9ORF72 repeat expansion was performed as described previously (Renton *et al.*, 2011) with minor modifications. Briefly, repeat-primed PCR was performed using 100 ng of DNA in a final volume of 28 µl containing (final concentrations): Roche (Indianapolis, IN) FastStart PCR Master Mix (1X), DMSO (7%, Sigma-Aldrich, St. Louis, MO), betaine (0.93M, Sigma-Aldrich, St. Louis, MO), deazaGTP (0.18 mM, Roche, Indianapolis, IN), MgCl_2_ (0.9 mM, Sigma-Aldrich, St. Louis, MO), and 10X primer mix (1X). The 10X primer mix was prepared containing 14 µM 6-FAM labeled forward primer (6-FAM-5‘AGTCGCTAGAGGCGAAAGC), 7 µM reverse repeat primer (5‘TACGCATCCCAGTTTGAGACGGGGGCCGGGGCCGGGGCCGGGG), and 14 µM anchor tail reverse primer (5‘TACGCATCCCAGTTTGAGACG). Touchdown PCR cycling conditions consisted of 4 min at 95°C followed by cycles of 95°C for 30 sec, annealing between 70°C–56°C for 1 minute, and extension at 72°C for 3 min, ending with a final extension step of 10 min at 72°C. The annealing temperature is decreased by 2°C in each step starting at 70°C for 2 cycles, 68°C for 3 cycles, 66°C for 4 cycles, 64°C for 5 cycles, 62°C for 6 cycles, 60°C for 7 cycles, 58°C for 8 cycles, and 56°C for 5 cycles. PCR product (2 µl) was mixed with 0.5 µl of ROX 500 Size Standard (Life Technologies, Carlsbad, California) and 7.5 µl Hi-Di formamide (Life Technologies, Carlsbad, California) and evaluated on an ABI 3130 capillary electrophoresis instrument with POP7 polymer and a 36 cm capillary with a 23 sec injection time. Interpretation of a positive expansion case was based on the presence of a stutter pattern while that of a case lacking the expansion produced one or more peaks with an abrupt ending peak. In some cases the absence of an expansion was confirmed using a standard 2 primer PCR reaction across the repeat region. The presence of 2 unique peaks was interpreted as negative for a repeat expansion while the identification of only a single peak was not informative. This 2 primer genotyping was performed using primers from by DeJesus-Hernandez et al with minor protocol modifications (Dejesus-Hernandez *et* *al.*, 2011). Briefly, the PCR was performed using 50 ng of DNA in a final volume of 20 µl containing (final concentrations): Amplitaq Gold buffer (1X), DMSO (5%), betaine (1 M), dNTP mixture with 7-deazaGTP instead of dGTP (0.25 mM each), MgCl_2_ (0.9 mM), forward and reverse primers (1 µM each), and Amplitaq Gold polymerase 0.5 U/reaction (Life Technologies, Carlsbad, California). PCR cycling conditions consisted of 10 min at 94°C followed by 36 cycles of 94°C for 35 sec, annealing between 62°C for 2 min, and extension at 72°C for 1 min, ending with a final extension step of 10 min at 72°C. The ABI 3130 electrophoresis conditions for this assay are the same as for the repeat-primed PCR reaction.

### Statistical Analysis

The “average” (and “range”) of data on patient characteristics was estimated by calculating the median (and 25th-75th percentiles). Differences between two groups were compared using Wilcoxon Mann-Whitney Test for quantitative and ordinal variables. To compare raw data of multiple groups, Kruskal-Wallis analysis of variance on ranks was applied, followed in case of significance by Dunn's Method in case. All correlations were studied using Spearman’s rank order correlation coefficient. Bonferroni-correction for multiple testing was applied when contrasts were not driven by a specific hypothesis. For all other tests, p-values <0.05 were considered significant. All statistical tests were 2-sided. A logistic ordinal regression model was used to test the association between the degree of microglial staining (dependant variable) and the presence of C9ORF72 expansions, adjusting for disease duration. Data analysis was performed using SPSS (Version 17.0 SPSS Inc., Chicago, IL, USA).

## Results

### Microglial Pathology as Depicted by CD68 and Iba1

To evaluate microglial pathology in the neuraxis of ALS, IHC with two different markers, CD68 and Iba1 was performed. Morphologically, activated microglial cells were observed to show a thin and elongated shape. In other reactive areas, a bushy and increasingly ramified morphology of microglial cells could be found (Figure 1). In the ventral horns of ALS spinal cord sections, an increased density of Iba1- and CD68-positive cells with enlarged cell processes, often in close proximity to motor neurons was observed. The density of ramified cells decreased in white matter regions containing the degenerating CST; instead, microglia transitioned to rounded macrophages (“myelinophages”) of varying sizes. These myelinophages were observed most extensively in the lateral and anterior cervical and lumbar CST of ALS, and where best visualized by CD68 IHC (Figure 1). Double-labeling IF showed incomplete co-localization of Iba1 and CD68 with Iba1 labeling mainly the cytoplasm and ramifications of microglia while CD68 mainly stained dot-like intracellular microglial compartments suggestive of endosomes/lysosomes, and phagosome-like profiles in macrophages (Figure S1).

**Figure 2:**
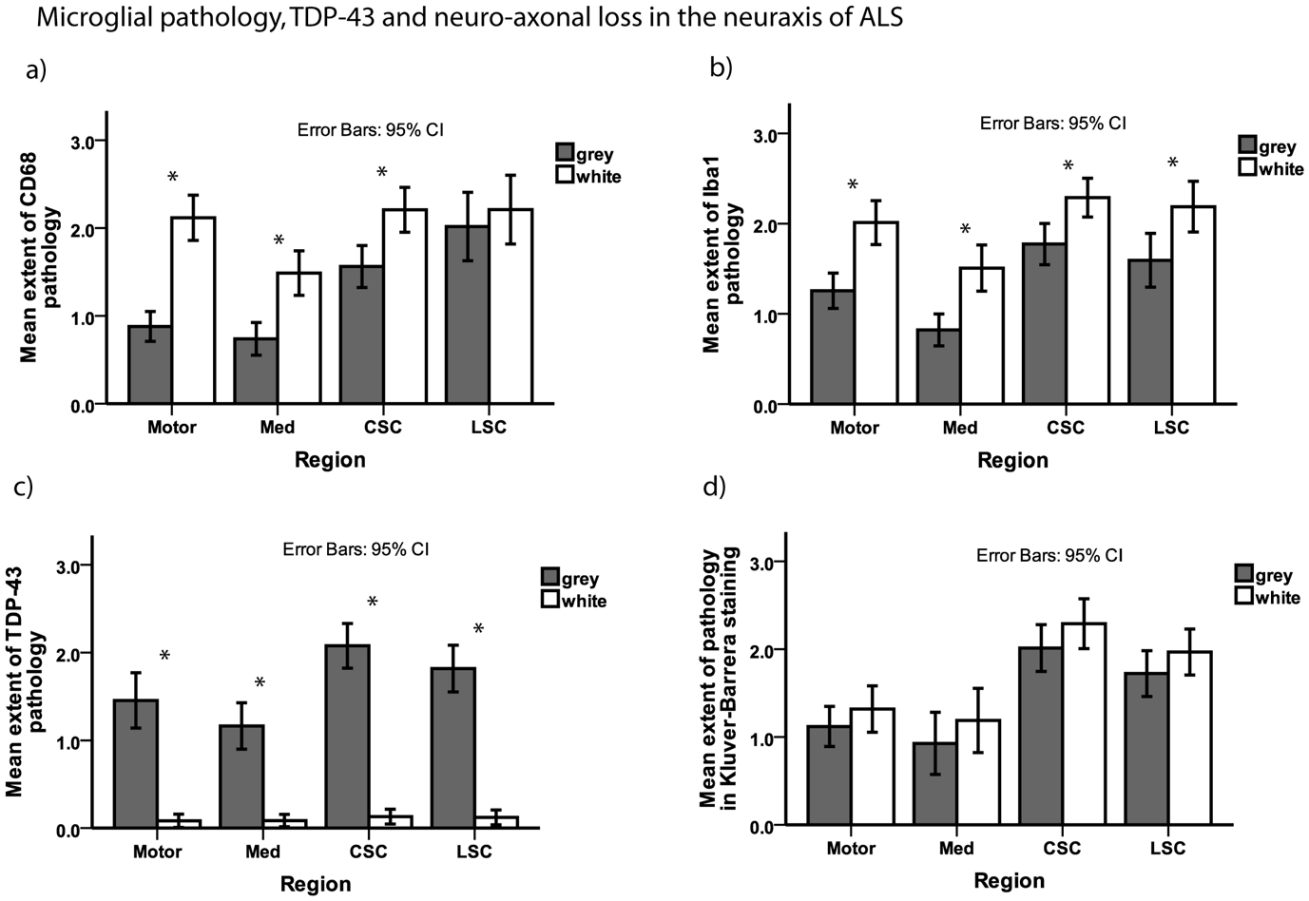
Microglial pathology, TDP-43, and neuro-axonal loss in the neuraxis of ALS. Bar plots show extent of microglial pathology as detected by staining for CD68 (a) and Iba1 (b) as well as TDP-43 pathology (c) and myelin loss as an indirect measure of axonal loss (d) in different regions of the neuraxis of ALS. For the spinal cord sections, the grey matter examined was the anterior horn and the white matter examined was the anterior and lateral portion of the CST. Whiskers in bar plot indicate 95% confidence interval of mean. CSC  =  cervical spinal cord, grey  =  grey matter, LSC  =  lumbar spinal cord, Med  =  medulla oblongata, motor  =  motor cortex (gyrus praecentralis), white  =  white matter.

Microglial pathology was most extensive in the cervical and lumbar CST (Figure 2). Neuronal loss was most extensive in cervical and lumbar anterior horns and axonal loss was most extensive in the cervical and lumbar CST which was reflected by IHC for CD68, Iba1 and TDP-43 as well as by myelin staining (Figure 2). Microglial pathology as measured by Iba1 correlated with axonal loss (as indirectly reflected by reductions in KB staining) in white matter subjacent to motor cortex (rho  = 0.40, p = 0.02, and rho  = 0.47, p = 0.01, respectively). The extent of microglial pathology as detected by both CD68 and Iba1 correlated with cervical CST axonal loss as reflected by reductions in KB staining and IHC for Nf proteins and MBP (rho >0.4, p<0.01 each). Microglial pathology as measured by Iba1 correlated with neuronal loss as measured by H&E staining of lumbar anterior horn sections (rho  = 0.4, p = 0.03).

TDP-43 pathology was observed throughout the grey matter of the neuraxis, with the most extensive TDP-43 positive inclusion pathology found in the anterior horn of the CSC (Figure 2). TDP-43 neuronal cytoplasmic inclusions varied in morphology from small granules and compact Lewy-body-like inclusions to filamentous skeins. In the neuraxis white matter analyzed here, only rare glial TDP-43 pathology was observed which was consistent with oligodendroglial TDP-43 inclusions. No correlation of TDP-43 pathology with neuronal or axonal loss as measured by any of the staining used here was observed in any of the regions analyzed (rho <0.4, p>0.05 each). The extent of microglial pathology and TDP-43 did not show a significant difference in any of the regions analyzed here between patients with sALS and fALS.

### Relation of Microglial Pathology and TDP-43 to Progression of Disease

**Figure 3:**
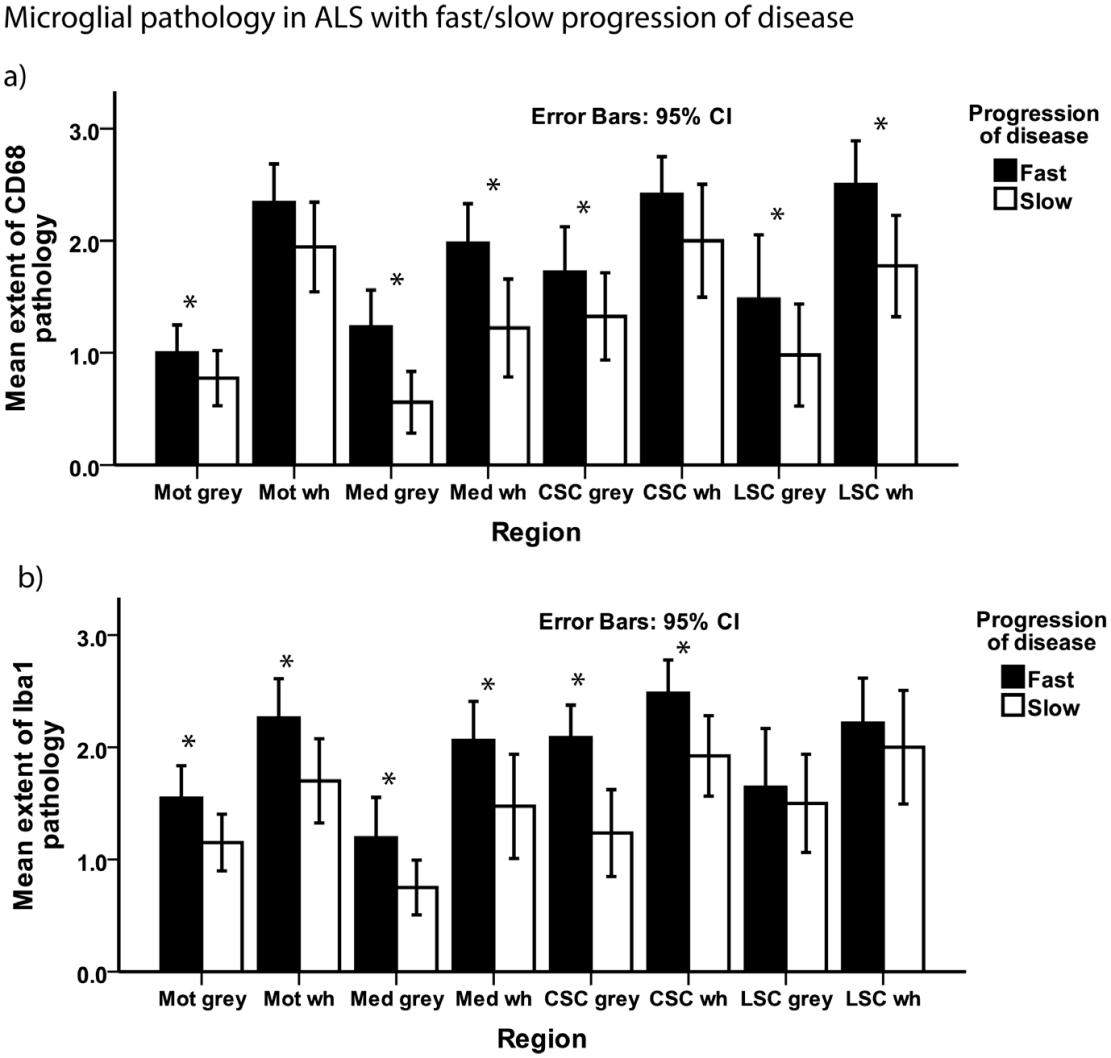
Microglial pathology and progression of disease. Bar plot shows microglial pathology as detected by staining for CD68 (a) and Iba1 (b) in ALS patients with a fast and a slow progression of disease. For the spinal cord sections, the grey matter examined was the anterior horn and the white matter examined was the anterior and lateral portion of the CST. Whiskers in bar plot indicate 95% confidence interval of mean. CSC  =  cervical spinal cord, grey  =  grey matter, LSC  =  lumbar spinal cord, Mot  =  motor cortex (gyrus praecentralis), Med  =  medulla oblongata, wh  =  white matter.

We next asked if microglial pathology was linked to the clinical phenotype including progression of disease. The median disease duration to death for the entire cohort of ALS autopsy cases was 24 months (Table 1). Patients with a disease duration shorter/equal to the median (n = 27) were defined as showing a rapid progression of disease [44], while cases with disease duration longer than 24 months (n = 26) were defined as showing a slow progression of disease (for 7 patients data on disease progression was missing). Cases with a rapid progression of disease showed more extensive microglial pathology as illustrated by CD68 IHC results in most of the regions analyzed here (p<0.05 each, Figure 3), with the exception of the CSC and motor cortex white matter, where the difference failed to reach statistical significance. Similarly, microglial pathology detected by Iba1 IHC was significantly more extensive in all areas analyzed here (p<0.05 each), with the exception of the LSC. TDP-43 pathology was significantly more extensive in the motor cortex of patients who showed a rapid progression of disease (p = 0.04), although no difference between patients with a fast and slow progression of disease was observed in any other region of the neuraxis analyzed here.

As we observed microglial pathology to correlate with disease progression, we next asked if it would also be associated with motor deficits as measured by clinical rating scales including a novel score of UMN deficits. The median UMN score for this cohort was 10 points (Table 1), with 25 patients scoring above the median, and 26 patients scoring below/equal to the median (for 9 patients, data on the UMN score was missing or incomplete). Patients with an UMN score above the median showed significantly more extensive microglial pathology in the cervical anterior horns and CST as detected by both CD68 and Iba1 when compared to patients who showed an UMN score below/equal to the median (p = 0.03 for each comparison). Furthermore, we observed higher scores on the UMN scale to correlate with more extensive microglial pathology in the cervical CST as detected by staining for both CD68 (rho = 0.54, p<0.01) and Iba1 (rho = 0.56, p<0.01). The UMN score also correlated with axonal loss as seen in KB staining in the cervical CST (rho  = 0.471, p = 0.007).

No significant differences regarding TDP-43 pathology were observed between patients with an UMN score above or below the median. No correlation of TDP-43 with microglial pathology was observed in any of the areas analyzed here. No correlation of the microglial markers (CD68, Iba1) or TDP-43 with the ALSFRS-R or the MRCS was observed. No correlation of postmortem interval to any of the markers analyzed here was observed.

### Microglial Pathology in ALS with/without *C9ORF72* Repeat Expansion

To identify ALS cases with a *C9ORF72* hexanucleotide repeat expansion in our cohort, we analyzed all sporadic and familial autopsy cases with ALS (n = 57) for which a DNA sample was available (DNA was not available for 2 cases). A*C9ORF72* hexanucleotide repeat expansion was identified in 15.8% (9/57) of ALS cases. For the subset of autopsy cases in which information about family history was known a *C9ORF72* expansion was identified in 27.2% (3/11) of ALS cases with a family history of ALS. The *C9ORF72* expansion rate was 25% (3/12) in ALS cases with a family history of neurodegenerative diseases other than ALS, and *C9ORF72* expansions were also identified in 9.4% (3/32) apparent sporadic ALS cases. The cohort included two with *SOD1* mutations, none of which were found to have a *C9ORF72* expansion.

Having identified nine ALS autopsy cases with a *C9ORF72* repeat expansion, we asked if the presence of this repeat expansion was linked to the clinical phenotype (Table 1). A bulbar onset of disease was significantly more frequent in the *C9ORF72* expansion ALS cases as compared to non-expansion cases (p = 0.04). Of 9 ALS cases with *C9ORF72* expansion, 5 (55.6%) showed a bulbar onset of disease, as compared to 26% in the subgroup of non-expansion cases. There was a tendency towards a shorter disease duration in the group with a *C9ORF72* repeat expansion, though this did not reach statistical significance (p = 0.08). ALS cases with *C9ORF72* repeat expansion showed a significantly higher ALSFRS-R as compared to non-expansion cases (p = 0.03). There was a tendency towards a higher MRCS in the expansion cases, though this did not reach significance (p = 0.09).

**Figure 4:**
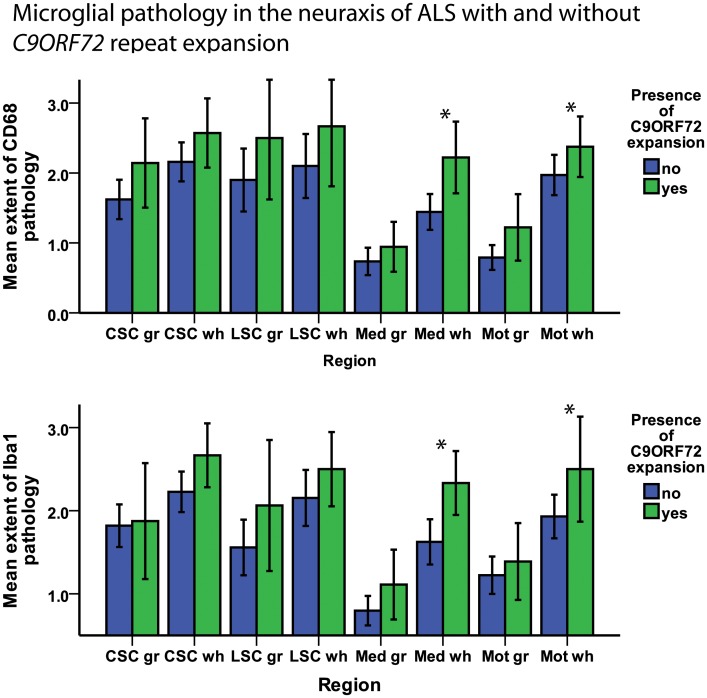
Microglial pathology in ALS with/without *C9ORF72* repeat expansion. Bar plot shows microglial pathology as detected by staining for CD68 (above) and Iba1 (below) in ALS patients with and without presence of a *C9ORF72* repeat expansion. For the spinal cord sections, the grey matter examined was the anterior horn and the white matter examined was the anterior and lateral portion of the CST. Whiskers in bar plot indicate 95% confidence interval of mean. CSC  =  cervical spinal cord, gr  =  grey matter, LSC  =  lumbar spinal cord, Mot  =  motor cortex (gyrus praecentralis), Med  =  medulla oblongata, wh  =  white.

As the presence of *C9ORF72* repeat expansions in ALS was associated with alterations in clinical phenotype, we next asked if this was associated with changes in microglial pathology. To determine the extent and regional distribution of microglial pathology in the neuraxis of ALS, the pathology labeled by CD68 and Iba1 was rated on a semi-quantitative scale. ALS cases with a *C9ORF72* repeat expansion showed a tendency towards more extensive microglial pathology in the grey and white matter of the neuraxis that reached statistical significance in the white matter of the medulla and the motor cortex (Figure 4). After adjusting for disease duration using logistic ordinal regression, the significance persisted for CD68 in the motor cortex (p = 0.04) and the medulla (p = 0.01) white matter as well as for Iba1 in the medulla white matter (p = 0.02), while it was lost for Iba1 in the motor cortex white matter (p = 0.11). No significant difference regarding the extent of TDP-43 pathology was observed between cases with and without *C9ORF72* repeat expansion in the regions analyzed here.

## Discussion

This study demonstrates that microglial pathology in the neuraxis of ALS patients correlates with disease progression, and shows it to be related to clinical UMN deficits, defining a novel clinical score to assess UMN damage that reflects CST pathology. It furthermore suggests that cases with a *C9ORF72* repeat expansion show more extensive microglial pathology in the motor cortex and the medulla as compared to non-expansion cases.

### Iba1 and CD68 as Markers of Microglial Activation in ALS

To analyze microglial pathology in the neuraxis of ALS, two different IHC markers, CD68 and Iba1 were applied. CD68 is a 110 kDa glycoprotein that is part of lysosomal membranes and shuttles in vesicles between lysosomes, endosomes, and the plasma membrane [45], [46]. CD68 is a pan-macrophage marker that is also expressed in microglia [45], [47]. The actin-binding protein IBA-1 is an established IHC marker for microglia that has been shown to be essential for membrane ruffling in response to macrophage colony-stimulating factor and phagocytosis in cultured cells, and is expressed on both resting as well as on activated microglia. [48]–[53]. We observed Iba1 to delineate ramified microglia in the neuraxis of ALS, whereas CD68 mainly stained intracellular microglial cell compartments suggestive of endosomes/lysosomes (Figure S1), indicating that Iba1 and CD68 could be of complementary usefulness as markers of microglial pathology in ALS.

### Microglial Pathology is Related to CST Axonal Loss in ALS

To quantify microglial pathology in different regions of the neuraxis of ALS, a semi-quantitative staging was applied. Our observation of extensive microglial pathology immunoreactive for CD68 and Iba1 confirms previous studies that demonstrated widespread microglial pathology in the spinal cord of ALS [54]–[56]. We observed the extent of microglial pathology to correlate with axonal loss in the CST. This supports the notion that proliferation and activation of microglia contributes to neurodegeneration in ALS, although it could merely be reactive to axonal and myelin loss. Microglia activated through the so-called classical pathway were shown to exert neurotoxic capacities through the secretion of reactive oxygen species and pro-inflammatory cytokines including TNF-α and IL-1β [57], [58], and mutant SOD1–expressing microglia were found to release higher levels of pro-inflammatory and cytotoxic cytokines in comparison to the wild-type microglia [59]. However, there is evidence that microglial activation in ALS could be a “double edged sword”, with microglial cells also showing neuroprotective capacities by releasing trophic and anti-inflammatory factors like IGF-1[60]–[62]. Therefore, microglia most likely have different phenotypic states and can exert both toxic and protective effects on motoneurons depending on the specific pathological conditions they encounter [17], [63].

### Microglial Pathology is Related to Progression of Disease and Clinical Signs of UMN Damage

Having observed microglial pathology to correlate with axonal loss in the CST, we next asked if it was also related to the clinical phenotype. While previous studies in rodents suggest an influence of microglia on progression of disease [64], [65], this has not been conclusively demonstrated in humans so far. Our data suggests that further understanding of the molecular pathology contributed to by microglial cells could help to develop disease-modifying therapies that reduce progression of disease benefiting patients with sporadic and familial ALS. In line with previous studies suggesting a deleterious loss of TDP-43 nuclear function could contribute to neuronal damage in ALS [2], [66], TDP-43 pathology was more extensive in the motor cortex of cases that showed a rapid progression of disease. However, no direct correlation of TDP-43 pathology with neuronal or axonal loss was shown here, which may be due to a clearance of TDP-43 from the extracellular space following its release from degenerating neurons harboring TDP-43 inclusions in the most severely affected regions of the end stage ALS nervous system.

Our observation that microglial pathology in ALS correlates with clinical signs of UMN damage is in line with observations of a PET study using a ligand binding to a mitochondrial benzodiazepine receptor that is activated in microglia showing microglial activation to be associated with the clinical UMN “burden” [28]. So far, there is no widely accepted clinical scoring system to assess UMN involvement in ALS [1], but we observed scores on the novel UMN rating scale used in this study to correlate with both axonal loss and microglial pathology in the CST. This study thereby provides basic observations on this scoring system to assess clinical UMN involvement that may validly reflect disease pathology in the CST of ALS. The lack of correlation between axonal loss and microglial pathology and the ALSFRS-R and the MRCS probably reflects the fact that these scales are more sensitive to lower motor neuron involvement and the resultant muscle wasting than UMN damage [1], [28].

### Microglial Pathology in ALS with *C9ORF72* Repeat Expansion

We next asked if the presence of *C9ORF72* repeat expansions in ALS would influence microglial pathology and clinical phenotype. Interestingly, and in line with a previous study [12], our subgroup of ALS with *C9ORF72* expansion showed a bulbar onset in over 50% of cases, as compared to a significantly lower frequency in the subgroup of non-expansion cases (26%). Generally, a bulbar onset of disease is clinically associated with speech and swallowing difficulties early-on in the disease, and is usually observed in approximately 25% of all ALS cases [1]. Our cases with *C9ORF72* expansion showed more extensive microglial pathology in the motor cortex and the medulla, while the difference as compared to non-expansion cases was not significant for other regions (Figure 4). It is so far unclear how *C9ORF72* hexanucleotide repeat expansion induces pathology and how this may in turn affect non-neuronal cells. In normal individuals, at least three alternatively spliced *C9ORF72* transcripts (variants 1–3) are expressed in the brain. Quantitative mRNA expression analysis indicated that the GGGGCC repeat expansion abolished *C9ORF72* transcript variant 1 expression, leading to an overall reduction in *C9ORF72* transcripts [11]. On a speculative level, changes caused by *C9ORF72* transcription could (through a yet unknown pathomechanism) be active further downstream in the pathological pathway of ALS, and influence inflammatory mechanisms linked to microglial pathology. However, the differences between *C9ORF72* expansion cases and non-expansion cases could also be partially due to the high proportion of bulbar-onset cases with a shorter disease duration in the expansion group, and could indicate reactive bulbar microglial activity in response to neuronal loss.

This study is limited by the lack of normal controls included here that do not allow to exclude that microglial alterations in ALS as assessed by CD68 and Iba1 may be partially due to post-mortem artifacts. However, given the statistical strength of the alterations observed here for a large cohort of patients with ALS, and previous studies by our group and others using the same markers [20], [67], [68], we still feel confident that the alterations observed here are valid and worthwhile relating.

## Supporting Information

Figure S1 Microglial pathology as shown by CD68 and Iba1. Double-labeling IF analyzed by confocal microscopy shows immunoreactivity of microglia for Iba1 (green) and CD68 (red) in the motocortex of an ALS autopsy case. While Iba1 depicts microglial cell morphology, CD68 mainly stains dot-like intracellular structures suggestive of endosomes/lysosomes; a) DAPI, b) CD68, c) Iba1, d) Merge/DAPI.
(TIF)

Table S1 Detailed description of upper motor neuron clinical score using a combined assessment of spasticity based on the Ashworth Spasticity Scale [32] with reflex scoring, and rating of pseudobulbar affect.
(DOC)
