# Supplementary figures and images for: Microglial Activation Correlates with Disease Progression and Upper Motor Neuron Clinical Symptoms in Amyotrophic Lateral Sclerosis
Source: PLoS One. 2012 Jun 14;7(6):e39216. doi: 10.1371/journal.pone.0039216 (PMC3375234; doi:10.1371/journal.pone.0039216)

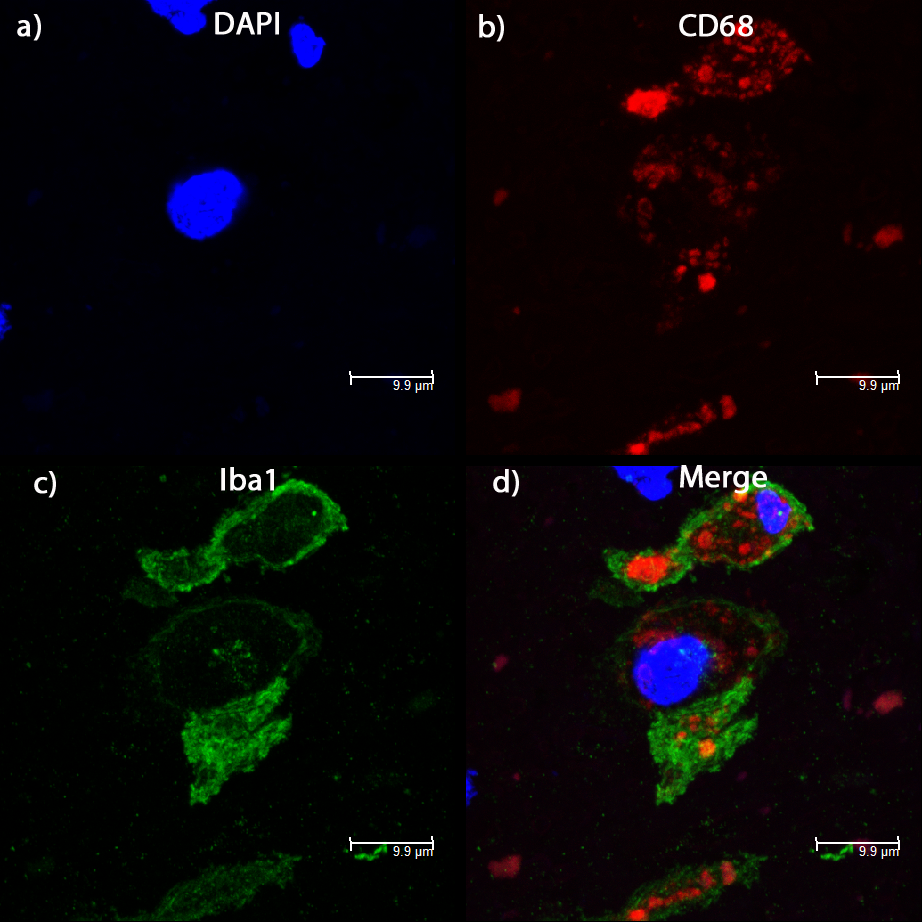

Supplement: Figure S1 — Microglial pathology as shown by CD68 and Iba1. Double-labeling IF analyzed by confocal microscopy shows immunoreactivity of microglia for Iba1 (green) and CD68 (red) in the motocortex of an ALS autopsy case. While Iba1 depicts microglial cell morphology, CD68 mainly stains dot-like intracellular structures suggestive of endosomes/lysosomes; a) DAPI, b) CD68, c) Iba1, d) Merge/DAPI. (TIF) [file pone.0039216.s001.tif]
