# Supplementary material for: Microglial Activation Correlates with Disease Progression and Upper Motor Neuron Clinical Symptoms in Amyotrophic Lateral Sclerosis
Source: PLoS One. 2012 Jun 14;7(6):e39216. doi: 10.1371/journal.pone.0039216 (PMC3375234; doi:10.1371/journal.pone.0039216)
Supplement: Table S1 — Detailed description of upper motor neuron clinical score using a combined assessment of spasticity based on the Ashworth Spasticity Scale [32] with reflex scoring, and rating of pseudobulbar affect. (DOC) [file pone.0039216.s002.doc]

# Upper Motor Neuron Score

Original Range: 0-33

Without thoracic segment included: 0-32

Spasticity Scoring:

Ashworth 1 = 0

Ashworth 2-3 = 1

Ashworth 4-5 = 2

Ashworth Spasticity Scale

1 = normal, no increase in tone

2 = slight increase in tone, giving a “catch” when affected part is moved

3 = more marked increase in tone, but affected part easily moved

4 = considerable increase in tone; passive movement difficult

5 = affected part rigid, immobile

Reflex Scoring:

Normal or absent = 0

Pathologically brisk = 1

Retained reflex in a weak or wasted limb = 1

Thoracic = 0 for present, 1 for absent

Pseudobulbar Affect Scoring:

CNS-LS 7-12 = 0

CNS-LS 13-35 = 1

(see below)

# UMN Score - score sheet

Bulbar

| Jaw Jerk (0-1) |  |
| --- | --- |
| Facial Reflex (0-1) |  |
| Palmomental Sign (0-1) |  |
| Pseudobulbar Affect (0-1, see below) | (CNS-LS ) |

## Right Cervical

| Spasticity (0-2) | (Ashworth ) |
| --- | --- |
| Triceps Reflex (0-1) |  |
| Biceps Reflex (0-1) |  |
| Finger Flexors (0-1) |  |
| Clonus (0-1) |  |
| Hoffman’s Sign (0-1) |  |

## Left Cervical

| Spasticity (0-2) | (Ashworth ) |
| --- | --- |
| Triceps Reflex (0-1) |  |
| Biceps Reflex (0-1) |  |
| Finger Flexors (0-1) |  |
| Clonus (0-1) |  |
| Hoffman’s Sign (0-1) |  |

## Thoracic

| Abdominal Reflex (0-1) |  |
| --- | --- |

## Right Lumbosacral

| Spasticity (0-2) | (Ashworth ) |
| --- | --- |
| Patellar Reflex (0-1) |  |
| Crossed Adduction (0-1) |  |
| Ankle Reflex (0-1) |  |
| Clonus (0-1) |  |
| Babinski Sign (0-1) |  |

## Left Lumbosacral

| Spasticity (0-2) | (Ashworth ) |
| --- | --- |
| Patellar Reflex (0-1) |  |
| Crossed Adduction (0-1) |  |
| Ankle Reflex (0-1) |  |
| Clonus (0-1) |  |
| Babinski Sign (0-1) |  |

**Pseudobulbar affect scoring**

Using the scale below, circle the number that describes the degree to which each item applies to you *DURING THE PAST WEEK*. Circle only one number for each item:

| Applies  Never | Applies  Rarely | Applies Occasionally | Applies Frequently | Applies Most of the Time |
| --- | --- | --- | --- | --- |
| 1 | 2 | 3 | 4 | 5 |

1. There are times when I feel fine one minute, and then I’ll become tearful the next over something small or for no reason at all.

1 2 3 4 5

2. Others have told me that I seem to become amused very easily or that I seem to become amused about things that really aren’t funny.

1 2 3 4 5

3. I find myself crying very easily.

1 2 3 4 5

4. I find that even when I try to control my laughter I am often unable to do so.

1 2 3 4 5

5. There are times when I won’t be thinking of anything happy or funny at all, but then I’ll suddenly be overcome by funny or happy thoughts.

1 2 3 4 5

6. I find that even when I try to control my crying I am often unable to do so.

1 2 3 4 5

7. I find that I am easily overcome by laughter.

1 2 3 4 5
